# Supplementary material for: Enhanced Bioactivity of Tailor-Made Glycolipid Enriched Manuka Honey
Source: Int J Mol Sci. 2022 Oct 10;23(19):12031. doi: 10.3390/ijms231912031 (PMC9570014; doi:10.3390/ijms231912031)
Supplement: Supplementary file 1 [file ijms-23-12031-s001.zip › ijms-1816264-supplementary.pdf]

Table S1. Stress assay reading at 20 hours displaying bioactivity of MH, MHWE, GOH, GDH, GLH and GMH at 0, 0.1, 0.2, 0.4, 0.8, 1.2 and 1.6 % (v/v). Fluorescence signals are normalized to the OD_{600} of the biosensor giving specific signals for each type of stress.

Mixture	Concentration % (v/v)	OD_{600}	RFP/OD_{600}	BFP/OD_{600}	GFP/OD_{600}
Control	0	1.01 ± 0.04	108 ± 3	1474 ± 223	694 ± 87
MH	0.1	1.02 ± 0.01	109 ± 12	1352 ± 65	239 ± 26
	0.2	0.74 ± 0.04	96 ± 20	1309 ± 55	234 ± 9
	0.4	0.74 ± 0.04	74 ± 11	1287 ± 36	233 ± 8
	0.8	0.76 ± 0.02	65 ± 13	1328 ± 33	185 ± 8
	1.2	0.78 ± 0.02	69 ± 14	1361 ± 37	190 ± 10
	1.6	0.77 ± 0.02	79 ± 6	1499 ± 40	202 ± 4
MHWE	0.1	1.03 ± 0.04	102 ± 18	1614 ± 155	279 ± 37
	0.2	0.85 ± 0.11	89 ± 9	1448 ± 80	180 ± 7
	0.4	0.84 ± 0.10	89 ± 7	1447 ± 50	173 ± 5
	0.8	0.82 ± 0.11	74 ± 8	1770 ± 129	148 ± 7
	1.2	0.84 ± 0.07	78 ± 8	1683 ± 83	149 ± 4
	1.6	0.87 ± 0.08	79 ± 9	1721 ± 101	147 ± 8
GOH	0.1	0.81 ± 0.04	126 ± 11	1449 ± 76	265 ± 17
	0.2	0.78 ± 0.09	121 ± 22	1169 ± 19	216 ± 5
	0.4	0.64 ± 0.02	127 ± 14	1360 ± 26	242 ± 7
	0.8	0.60 ± 0.02	220 ± 15	1578 ± 11	303 ± 4
	1.2	0.69 ± 0.02	582 ± 47	1855 ± 95	313 ± 11
	1.6	0.66 ± 0.08	589 ± 28	2512 ± 51	500 ± 38
GDH	0.1	1.00 ± 0.02	79 ± 13	1429 ± 129	284 ± 27
	0.2	0.93 ± 0.03	91 ± 21	1425 ± 187	176 ± 4
	0.4	0.80 ± 0.03	79 ± 7	1398 ± 109	160 ± 6
	0.8	0.85 ± 0.08	80 ± 13	1674 ± 359	158 ± 5
	1.2	1.10 ± 0.07	76 ± 18	1507 ± 380	132 ± 6
	1.6	1.19 ± 0.04	75 ± 13	1269 ± 187	123 ± 5
GLH	0.1	1.04 ± 0.01	206 ± 10	1335 ± 34	283 ± 31
	0.2	1.01 ± 0.03	178 ± 16	1331 ± 61	168 ± 13
	0.4	0.81 ± 0.01	83 ± 5	1174 ± 46	185 ± 5
	0.8	1.00 ± 0.02	99 ± 9	1054 ± 20	132 ± 2
	1.2	1.07 ± 0.03	99 ± 9	1064 ± 42	126 ± 8
	1.6	1.11 ± 0.02	106 ± 7	1101 ± 37	127 ± 5
GMH	0.1	1.17 ± 0.03	142 ± 39	1806 ± 247	337 ± 100
	0.2	0.78 ± 0.02	146 ± 20	1739 ± 29	219 ± 4
	0.4	0.72 ± 0.03	140 ± 27	1803 ± 78	227 ± 6
	0.8	0.80 ± 0.05	103 ± 9	1623 ± 91	189 ± 5
	1.2	0.84 ± 0.05	123 ± 29	1791 ± 149	196 ± 7
	1.6	0.91 ± 0.08	157 ± 24	1887 ± 268	201 ± 7
